# News and Coming Events

**Published:** 1909-03-06

**Authors:** 


### News and Coming Events.

Dr. J. R. Phillips, of the Colonial Civil Service, has been appointed a member of the Legislative Council of Barbadoes.

Dr. Stanley Colyer, M.D., B.S.Lond., M.R.C.P. Lond., has been appointed Honorary Medical Officer to the Royal Halifax Infirmary.

Mr. A. Logan Murison, M.R.C.S., L.R.C.P.Lond., has been appointed Assistant Surgeon to the Metropolitan Ear, Nose, and Throat Hospital.

Mr. Edward W. Bain, M.B., B.S.Lond., F.R.C.S.Eng., has been appointed Assistant Surgeon to the Metropolitan Ear, Nose, and Throat Hospital.

Dr. Donald J. Munro, M.B.Lond., M.R.C.S., L.R.C.P.Lond., has been appointed Anæsthetist to the Metropolitan Ear, Nose, and Throat Hospital.

Dr. Gordon Holmes, M.D., B.Ch., B.A.O.Dub., has been appointed Assistant Physician to Out-patients at the National Hospital for the Paralysed and Epileptic.

Dr. G. D. Freer, who for the past eighteen years has occupied various medical offices in the Straits Settlements and the Federated Malay States, has been appointed senior medical officer, Selangor, Federated Malay States.

The annual general meeting of the National Hospital for the Relief and Cure of the Paralysed and Epileptic (Albany Memorial) will be held at the Hospital, Queen Square, Bloomsbury, W.C., on Tuesday, March 16, 1909, at 3 p.m.

The annual general meeting of the North London or University College Hospital, Gower Street, W.C., will be held on Thursday, March 25 in the board-room of the Hospital at 4 p.m. precisely.

The first case of small-pox in London since August 1907 is reported to have occurred in Camberwell. The patient had been working in Egypt up to January 15, and it is supposed that he was infected while on the voyage home.

Dr. Frederick Taylor has been appointed the representative of the University of London on the General Medical Council, in the place of Dr. P. H. Pye-Smith, F.R.S., resigned. The degree of D.Sc. in Physiology has been awarded by the University to Dr. F. H. Scott, Ph.D., M.B., an internal student, of University College, for a thesis entitled "On the Relative Parts Played by Nervous and Chemical Factors in the Regulation of Respiration," and other papers.

The Hon. W. F. D. Smith, M.P., presided at the Court of the Corporation of King's College Hospital, held on February 25, and moved the adoption of the seventieth annual report of the Committee of Management. The number of in-patients during the past year was 2,677, as against 2,682 in 1907, while the number of cases treated in the out-patient department was 511 fewer than in the previous year. There was an increase in ordinary income of £2,854, mainly due to an increased grant from King Edward's Hospital Fund and to an increase of £576 under the head of donations. The annual subscriptions were £65 less. Legacies amounted to £15,779. The ordinary expenditure, although £498 less than the previous year, exceeded the ordinary income by £3,953, and in order to meet this deficiency, together with the debt of the previous year, it was necessary to utilise for current purposes the major portion of the legacies received.

The annual general meeting of Governors of the Samaritan Free Hospital for Women, Marylebone Road, N.W., will be held in the board-room on Tuesday, March 9, at 5 p.m., to receive the report and cash statement for 1908, and to elect the Committee for the ensuing year.

Dr. H. W. King, a well-known Chester medical practitioner, died last week, after a short illness, at the age of fifty-three. He was educated at Edinburgh University, and graduated M.B. in 1878, subsequently taking the M.D. of that University and the M.R.C.S. England. Dr. King was honorary physician to the Chester General Infirmary.

The annual general meeting of the Governors of the London Fever Hospital, Liverpool Road, N., was held last week at the Hotel Victoria, under the chairmanship of Lord Balfour. Dr. Sidney Phillips, the senior physician, read the report, which showed that the total number of patients under treatment during the year was 762, or 90 less than in the previous year, the decrease being due entirely to the lessened number of cases of German measles admitted. Sir Thomas Barlow, in moving the adoption of the report, urged the establishment of voluntary pay hospitals throughout the country.

The London Ophthalmic Exhibition will be held on March 12 and 13, from 12 noon to 10 p.m. each day, at the rooms of the Medical Society of London, 11 Chandos Street, Cavendish Square, London, W. A number of optical firms of repute are showing the latest optical and ophthalmological appliances. The object of the London Ophthalmic Exhibition is to bring under the notice of medical men, and especially of ophthalmic surgeons the latest models and improvements that have been invented during the year. Tickets will be sent free on application to the Organising Secretary, Ernest Schofield, 11 Chandos Street, Cavendish Square, London, W.

The following lectures will be delivered at the Royal College of Physicians of London, Pall Mall East: The Milroy lectures, by Dr. R. Tanner Hewlett, on March 2, 4, and 9, on "Disinfection and Disinfectants"; the Goulstonian lectures, by Dr. A. E. Russell, on March 11, 16, and 18, on "Some Disorders of the Cerebral Circulation and their Clinical Manifestations"; the Lumleian lectures, by Dr. Norman Moore, on March 23, 25, and 30, on "Rheumatic Fever and Valvular Disease"; and the Oliver-Sharpley lectures, by Professor C. S. Sherrington, M. D., F.R.S., on April 1 and 2, on "The Rôle of Reflex Inhibition in the Co-ordination of Muscular Action." The lectures will commence at five o'clock p.m.

The Lord Mayor will preside at the 81st Annual Meeting of the Royal Free Hospital, Gray's Inn Road, London, W.C., to be held at the Mansion House on Wednesday, March 10 next, at 3 p.m., when the claims of the hospital for increased public support will be advocated. The following are expected to take part in the proceedings: The Earl of Sandwich (Chairman of the Committee), the Bishop of Stepney, Sir Henry C. Burdett, K.C.B., K.C.V.O., Sir Edwin Durning-Lawrence, Bart., Sir F. Layland Barratt, Bart., M.P., W. H. Dickenson, Esq., M.P., the Mayor of St. Pancras, and Mrs. Scharlieb, M.S. The Secretary will, upon application, forward cards of invitation to the Mansion House Meeting, or will gladly acknowledge any contributions to be announced on that occasion.

